# Methamphetamine-associated psychosis: a new health challenge in Iran

**DOI:** 10.1186/2008-2231-21-30

**Published:** 2013-04-11

**Authors:** Zahra Alam mehrjerdi, Alasdair M Barr, Alireza Noroozi

**Affiliations:** 1 Iranian National Center for Addiction Studies (INCAS), Tehran University of Medical Sciences, No.669, South Karegar Ave, Tehran, Iran; 2 Department of Anesthesiology, Pharmacology & Therapeutics, University of British Columbia, Vancouver, BC, Canada; 3 Iranian National Center for Addiction Studies (INCAS), School of Advanced Technologies in Medicine (SATM), Tehran University of Medical Sciences, Tehran, Iran

## Abstract

The rapidly growing popularity of methamphetamine use in Iran has posed a new health challenge to the Iranian health sector. Methamphetamine-associated psychosis (MAP) has been frequently reported in Iran in recent years. Although methamphetamine use and MAP are considerable health problems in Iran but there is still a need to conduct epidemiological studies on the prevalence of MAP and its health-related problems. The present paper emphasizes that health policy makers should consider the immediate needs of drug users, their families and the community to be informed about the detrimental health effects associated with MAP. Although MAP could be managed by prescribing benzodiazepines and psychiatric medications but the most effective regime for stabilizing patients with MAP still needs to be studied in Iran. Constant collaborations among psychiatric services and outpatient psychotherapeutic services should be established to successfully manage MAP in Iran. Iranian clinicians especially emergency medicine specialists should be informed about the differences between the two forms of transient and recurrent MAP in order to implement appropriate pharmacological therapies to manage MAP. It is hoped that special training courses are designed and implemented by health policy makers to inform clinicians, health providers and especially emergency medicine specialists to effectively deal with MAP.

## Editorial

For centuries, opium and opium residues have been used by Iranians but in recent years, methamphetamine (MA) use has emerged as an epidemic health concern among Iranian substance users [1]. Methamphetamine is known as a synthetic derivative of amphetamine, but due to the addition of a methyl group in its chemical structure, it has lipid solubility, allowing more rapid transport of the drug across the blood–brain barrier [2].

Methamphetamine use and abuse can result in methamphetamine**-**associated psychosis (MAP) which has a recurrent nature [3]. MAP is characterized by auditory or visual hallucinations, ideas of reference, persecutory delusions, thought reading, strange or unusual beliefs, delusions of reference and thought insertion [4]. The incidence and severity of MAP are related to high doses of methamphetamine use, the routes of administration, and duration of regular use [5,6]. MAP is more likely to occur in methamphetamine patients even after adjusting with psychotic disorders [7,8]. The presence of MAP is generally transient and normally abates in a few days. However, susceptibility to psychotic episodes could prolong for years after prevention from methamphetamine use [3]. Studies show that methamphetamine use [3], using other drugs [9] and psychosocial stressors [10] could trigger the relapse of MAP. MAP is also associated with considerable health service utilization and increased psychiatric symptoms over time [11]. Chronic MAP is persistent and so similar to those of schizophrenia. Studies show that susceptibility to MAP could last for years after preventing from methamphetamine use [5].

Currently, methamphetamine use has rapidly emerged and developed in Iran [12,13]. A recent rapid situational assessment (RSA) of substance use showed that only a small group of Iranian substance users (3.6%) reported methamphetamine as their main drug of abuse [14] but in the past several years, the price of methamphetamine has dramatically decreased. Current nonofficial estimates suggest that methamphetamine is the second or third illicit drug in Iran [14] and an increasing number of patients with MAP are admitted to Iranian psychiatric hospitals [15].

In a study on the symptoms and treatment of MAP during one**-**year follow**-**up, researchers studied a 38 year**-**old man who was admitted to the emergency department setting of Iranian educational and therapeutic center of psychiatry because of suicide attempts, auditory hallucinations and persecutory delusions. The patient was treated with prescribing antipsychotic medications and experienced a short recurrence of MAP during abstaining from methamphetamine use, as well as a MAP episode although he was receiving antipsychotic medications [16].

Omidvar and Sharifi (2012) examined a patient who was diagnosed with MAP and eye autoenucleation. The patient had delusions of being in control and believed that others were using his eyes. He was diagnosed with a first episode of MAP and did not report MAP symptoms in the absence of methamphetamine use but he was treated with six sessions of electroconvulsive therapy (ECT) and prescribing psychiatric medications [17]. A study on 50 methamphetamine abusers recruited from the Iranian legal medical organization in Tehran showed that delusion, mood instability, disorientation, and self-mutilation were prevalent among the patients [18].

In another study, the clinical files of 111 methamphetamine patients who had been admitted to a central hospital in Tehran from April 2008 to April 2010 were reviewed. The study results showed that the most prevalent psychotic symptoms were persecutory delusions (82%), auditory hallucinations (70.3%), reference delusions (57.7%), visual hallucinations (44.1%), grandiosity delusions (39.6%) and jealousy delusions (26.1%). The mean duration of admission and psychotic episodes were 21.43 and 17.37 days respectively. In seven cases (8.75%), MAP continued for more than one month [19]. Methamphetamine**-**related health problems such as MAP have changed the profile of patients who utilize psychiatric services in Iran [20]. Because of heavy use, many of such cases are characterized by delusions, hallucinations, anxiety, insomnia, mood disturbances, suicide, violent behaviors and homicidal ideations [20]. This is consistent with some studies in other countries which indicate that heavy use of methamphetamine contributes to a variety of severe psychiatric problems including MAP [21].

It should be noted that the rapidly growing popularity of methamphetamine use has posed a new health challenge to the Iranian health sector. MAP is associated with a range of acute and chronic health and social problems that need to be considered during the course of treatment especially in emergency department settings, and psychiatric and general hospitals. In order to effectively deal with MAP in Iran, several issues should be considered by health policy makers. Although MAP is prevalent among methamphetamine users in Iran, epidemiological studies on the prevalence of MAP and its health**-**related problems should be conducted.

The negative effects of MAP on methamphetamine patients, and the community should be considered by health policy makers. Nationwide drug education should be designed and implemented to inform drug users, their families and the community about the harms associated with MAP and the necessity of immediate referrals of such cases to emergency department settings of psychiatric hospitals. MAP could be treated with prescribing psychiatric medications and benzodiazepines in a supportive non**-**judgmental relationship. Atypical antipsychotics have become first line treatments for psychotic disorders, especially first episode psychosis [22]. Recently, case reports of successful MAP treatment with atypical antipsychotics such as Olanzapine [23], Risperidone [24] and Quetiapine [25] have been reported. Such studies have been conducted in other countries such as USA and Australia. There is an immediate need for Iran to devise a treatment protocol to pharmacologically manage MAP according to its health and medical**-**related conditions.

Little is known about the pharmacological therapies of patients with MAP in Iran. Moreover, the most effective regime for stabilizing patients with MAP still needs to be studied in Iran. Once, the pharmacological therapy of MAP is implemented in a psychiatric hospital, referring patients to outpatient drug use treatment clinics is implemented. In many instances, the involvements of such services include outpatient psychotherapeutic services and there is still a need to establish constant collaborations among psychiatric services and outpatient psychotherapeutic services to successfully manage MAP.

Methamphetamine users are vulnerable to MAP, either from exacerbation of symptoms of underlying psychotic disorders [26] or emerging new psychotic symptoms during intoxication and withdrawal stages [27] and recurrence can occur in response to psychological stressors even in the absence of methamphetamine use [28].

Iranian clinicians should know that psychotic symptoms tend to persist in some patients. Therefore, the distinction between persistent MAP and a primary transient MAP has grown increasingly important. Iranian clinicians especially emergency medicine specialists should be informed about the differences between these two forms of MAP and recurrence of MAP in order to implement appropriate pharmacological therapies to manage MAP. Primary and emergency health care providers, mental health and substance abuse treatment service providers need to be trained to distinguish between MAP, other forms of toxic psychosis, and primary psychosis. A further challenge to primary and emergency health care service providers lies in the detection and management of transient MAP and persistent MAP. This issue needs attention by health policy makers.

To sum up, as MAP negatively influences Iran, new nationwide and research**-**oriented strategies should be designed and implemented to meet the increasing needs of the community, and health care providers especially emergency medicine specialists to effectively deal with MAP. Given the challenges that MAP has posed to health professionals and the community, the role of the health sector in the provision of evidence**-**based treatment for MAP is emphasized. In order to fulfill this role, it is important that the health sector such as the medical schools also provides health care professionals with evidence**-**based information and training related to the diagnosis and management of MAP.

## Authors’ contributions

ZAM wrote the manuscript. AMB and AR N provided editorial assistance and scientific advice. All authors read and approved the final manuscript.

## Financial disclosure

None.
